# Squamous Metaplasia of the Colon Following Severe COVID-19

**DOI:** 10.7759/cureus.24105

**Published:** 2022-04-13

**Authors:** Satoshi Masuda, Taiki Aoyama, Mayumi Kaneko, Shinji Nagata

**Affiliations:** 1 Gastroenterology, Hiroshima City Asa Citizens Hospital, Hiroshima, JPN; 2 Diagnostic Pathology, Hiroshima City Asa Citizens Hospital, Hiroshima, JPN

**Keywords:** colon, coronavirus, endoscopy, colonoscopy, diarrhea

## Abstract

A 53-year-old man was admitted for respiratory failure due to severe acute respiratory syndrome caused by a severe acute respiratory syndrome coronavirus 2 infection. The patient required prolonged artificial ventilation and extracorporeal membrane oxygenation (ECMO) for respiratory support. Despite successful discontinuation of ECMO, the patient experienced profuse watery diarrhea (5-10 L/day). A colonoscopy revealed an inflamed surface without undulation that uniformly extended throughout the colon. Biopsy specimens revealed complete disappearance of existing crypts and replacement with squamous or transitional epithelium normally observed in the anal transitional zone mucosa, with granulation tissue proliferation in the lamina propria. Watery diarrhea persisted despite corticosteroid and infliximab administration. Although diarrhea due to atrophy of the surface and cryptic epithelium as an intestinal manifestation of coronavirus disease 2019 usually responds to corticosteroids, refractory diarrhea can be attributed to squamous metaplasia with complete disappearance of the surface and cryptic epithelium.

## Introduction

An association has been reported between severe coronavirus disease 2019 (COVID-19) and gastrointestinal symptoms such as diarrhea and abdominal pain [1,2]. Although some patients with severe COVID-19 experience refractory diarrhea, only a few reports have revealed endoscopic findings for investigation of the etiology. Endoscopic findings such as mucosal detachment and edematous mucosa resembling graft-versus-host disease have been reported, along with pathological findings such as inflammatory cell infiltration and atrophy of the glandular fossa [3,4]. Such cases can respond to treatment with corticosteroids with or without infliximab and fluid resuscitation, and, consequently, diarrhea can be resolved [3,4].

Here, we report a case of severe COVID-19 presenting with refractory diarrhea, with endoscopic findings in the colon resembling those of previous reports but with differing histopathological findings.

## Case presentation

**Figure 1:**
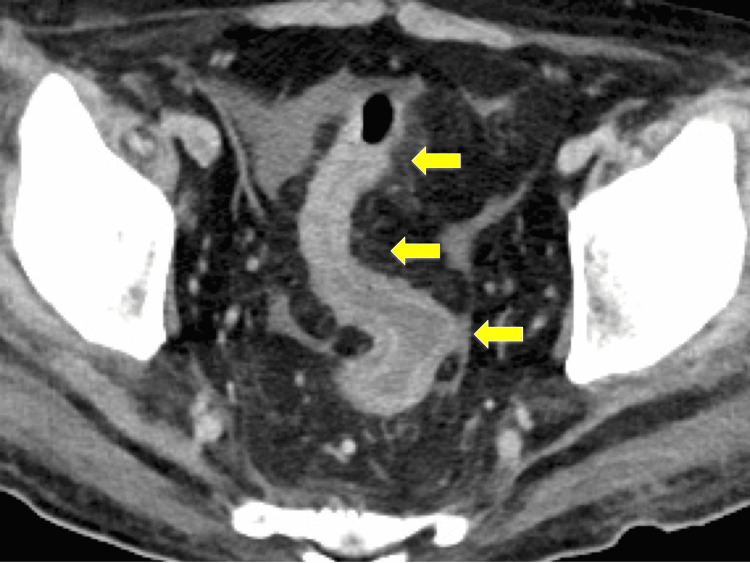
Contrast-enhanced computed tomography. Computed tomography showing edematous changes throughout the colon (arrows).

**Figure 2:**
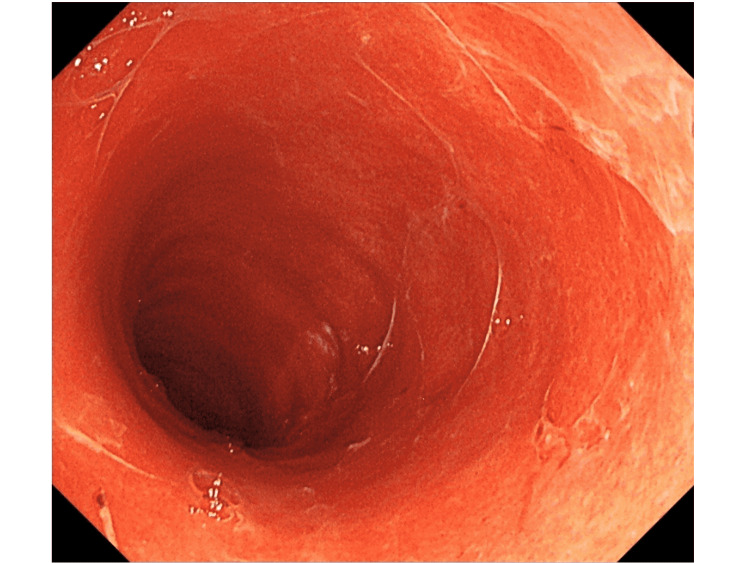
Colonoscopic image (rectum). Colonoscopy showing diffusely inflamed colonic mucosa without undulation, with mucus attached to the surface.

**Figure 3:**
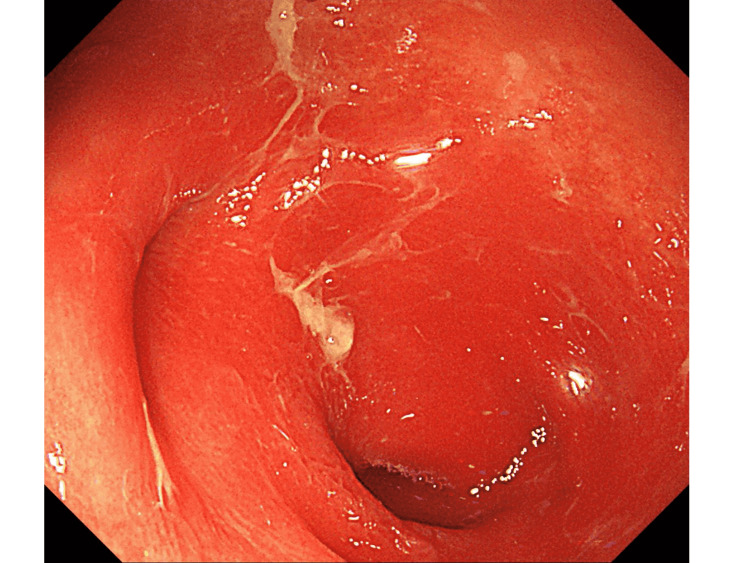
Colonoscopic image (cecum). Colonoscopy showing diffusely inflamed colonic mucosa without undulation, with mucus attached to the surface.

In spring 2021, a 53-year-old man with a history of hypertension was admitted for hypoxemic respiratory failure due to a severe acute respiratory syndrome coronavirus 2 (SARS-CoV-2) infection. Computed tomography (CT) revealed diffuse ground-glass opacities in the bilateral lobes of the lung. Despite multidisciplinary treatment, including steroid use, he developed respiratory failure within one week and required prolonged artificial ventilation for 51 days and extracorporeal membrane oxygenation (ECMO) for seven days for respiratory support. Despite the successful withdrawal from ECMO, a week after discontinuation, the patient experienced profuse watery diarrhea (5-10 L/day). Although causative medicines that could lead to intestinal damage were not administered, the stool culture results were negative for *Clostridium difficile* or other pathogens. Supplementation of extracellular fluid equivalent to the amount of diarrhea was required to maintain circulatory dynamics. A repeat contrast-enhanced CT examination showed edematous changes throughout the colon (Figure 1); however, these were not remarkable in the small intestine. Presumptive intestinal ischemia or a thrombus in the abdominal vessels was not observed. Watery diarrhea persisted despite corticosteroid, antidiarrheal, and mesalazine administrations. A colonoscopy performed to determine the etiology of the disease revealed an inflamed surface without colonic folds, uniformly extending throughout the colon (Figures 2, 3).

**Figure 4:**
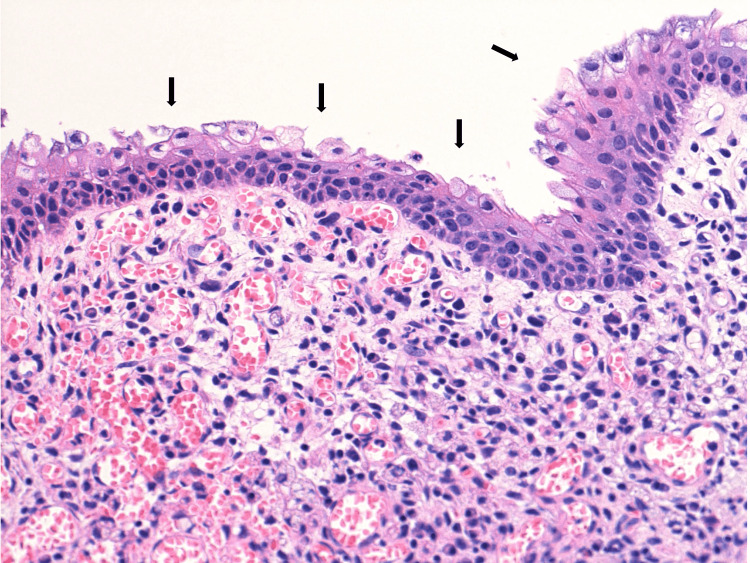
Pathology. Histopathological findings show the disappearance of crypts and complete replacement with squamous or transitional epithelium in the colonic mucosa (arrows).

The mucosa was completely detached. Biopsy specimens taken from relevant mucosa revealed the complete disappearance of existing crypts and replacement with squamous or transitional epithelium normally seen in the anal transitional zone mucosa, with granulation tissue proliferation in the lamina propria (Figure 4).

The immunochemical examination revealed that cytomegalovirus (CMV) infection was unlikely. Polymerase chain reaction testing of the colonic mucosa for the SARS-CoV-2 virus yielded negative results. The endoscopic and pathologic findings showed no abnormal changes on esophagogastroduodenoscopy, while the entire small intestine was not endoscopically explored. Watery diarrhea persisted despite the administration of corticosteroids (1 mg/kg) and infliximab (5 mg/kg). The patient died of multiple organ failure five months after the onset of diarrhea.

## Discussion

SARS-CoV-2 enters the intestinal mucosa and causes gastrointestinal symptoms such as diarrhea or abdominal pain. According to one meta-analysis from China, the frequency of gastrointestinal symptoms is reported to be 17.6%, which correlates with the severity of respiratory symptoms [2]. The putative mechanism underlying the development of gastrointestinal symptoms in COVID-19 patients involves a viral cytopathic effect with or without subsequent intestinal inflammation [4]. Meanwhile, “long-COVID” syndrome is conceptualized as prolonged or late-onset sequelae based on multiorgan disorders with a wide spectrum of clinical manifestations, including gastrointestinal disorders [5,6]. Systemic inflammation and oxidative stress processes are thought to prevail as mechanisms of “long-COVID” syndrome [7]. Thus, gastrointestinal manifestations can occur in any phase of the disease.

Some cases of diarrhea in COVID-19 have been attributed to CMV infection [4,8,9]. This may be due to immune dysregulation during severe COVID-19 pneumonia or the use of immunosuppressants [8]. Diarrhea induced by CMV infection is usually resolved with anti-CMV treatment [8,9]. However, the immunochemistry examination did not reveal CMV infection in our case.

A few reports have revealed endoscopic and histopathologic findings in patients with COVID-19 presenting with profuse diarrhea. Endoscopic findings such as extensive mucosal sloughing in the small intestine and colon have been reported [3,4]. In addition, histopathological findings such as mucosal erosion, crypt loss, edema, lymphocyte infiltration, and enterocyte apoptosis have been reported [3,4]. Similar to that in previous reports, colonoscopy showed completely detached mucosa throughout the colon in our case. Histopathological findings revealed inflammatory cell infiltration in the lamina propria, similar to those in previous reports [3,4]. However, the findings on the surface were completely different. Complete disappearance of existing crypts and replacement with squamous or transitional epithelium, normally seen in the anal transitional zone mucosa, were found in our case. In colorectal tissue, squamous metaplasia arises in polypoid lesions, including adenomas or adenocarcinomas, with a frequency of 0.4%, and this finding can be focally observed in these lesions [10-12]. Squamous metaplasia in the colon subsequent to inflammation has been reported to exist in patches or segments [13,14]. The background of diseases may affect macroscopic findings. However, complete replacement of the colorectal mucosa with squamous or transitional cell metaplasia of the entire colon has never been reported. Although it has been reported using autopsy specimens that squamous metaplasia develops in the lung after the second week of symptom onset in patients with severe COVID-19 [15], whether the same mechanism of squamous metaplasia in the lung can be applied to the colon remains unclear. We posit that this histopathologic result may be due to the complex incorporation of prolonged systemic inflammation, viral cytopathic effect, oxidant stress, stimulation from ECMO, or unknown processes.

Diarrhea due to atrophy of the surface and cryptic epithelium as an intestinal manifestation of COVID-19 responds to corticosteroids [3,4] and shows remarkable improvement when infliximab is used [3]. However, our patient was refractory to the existing treatments. The difference in response to the treatment can be attributed to the difference in the surface: squamous metaplasia with complete disappearance of the surface and the cryptic epithelium. Histopathological assessment and endoscopic surveys are required to determine the effectiveness of these treatments.

## Conclusions

This case demonstrated an intestinal complication of COVID-19 presenting with refractory diarrhea. Colonoscopy was useful for investigating the etiology. In the colon, pathology, including complete disappearance of existing crypts and replacement with squamous or transitional epithelium, may be resistant to existing treatments and deliver unfavorable outcomes.
